# Optimal extraction, purification and antioxidant activity of total flavonoids from endophytic fungi of *Conyza blinii* H. Lév

**DOI:** 10.7717/peerj.11223

**Published:** 2021-04-09

**Authors:** Shuheng Zhao, Xulong Wu, Xiaoyu Duan, Caixia Zhou, Zhiqiao Zhao, Hui Chen, Zizhong Tang, Yujun Wan, Yirong Xiao, Hong Chen

**Affiliations:** 1 College of Life Sciences, Sichuan Agricultural University, Ya’an, China; 2 Chengdu Agricultural College, Chengdu, China; 3 Sichuan Food Fermentation Industry Research and Design Institute, Chengdu, China; 4 Sichuan Agricultural University Hospital, Ya’an, China; 5 College of Food Sciences, Sichuan Agricultural University, Ya’an, China

**Keywords:** Chaetomium cruentum, Response surface, Macroporous resin, Flavonoid

## Abstract

**Background:**

Flavonoids are widely used in the market because of their antibacterial, antiviral, and antioxidant activities. But the production speed of flavonoids is limited by the growth of plants. CBL9 (*Chaetomium cruentum*) is a flavonoid-producing endophytic fungi from *Conyza blinii* H. Lév, which has potential to produce flavonoids.

**Methods:**

In this study, we isolated total flavonoids from endophytic fungus CBL9 of *Conyza blinii* H. Lév using macroporous resin D101. The process was optimized by response surface and the best extraction process was obtained. The antioxidant activities of total flavonoids were analyzed in vitro.

**Results:**

It was found that the best parameters were 25 °C pH 2.80, 1.85 h, and the adsorption ratio reached (64.14 ± 0.04)%. A total of 60% ethanol was the best elution solvent. The elution ratio of total flavonoid reached to (81.54 ± 0.03)%, and the purity was 7.13%, which was increased by 14.55 times compared with the original fermentation broth. Moreover its purity could rise to 13.69% after precipitated by ethanol, which is very close to 14.10% prepared by ethyl acetate extraction. In the antioxidant research, the clearance ratio of L9F-M on DPPH, ABTS, •OH, •O^2−^, (96.44 ± 0.04)% and (75.33 ± 0.03)%, (73.79 ± 0.02)%, (31.14 ± 0.01)% at maximum mass concentration, was higher than L9F.

**Conclusion:**

The result indicated using macroporous resin in the extraction of total flavonoid from endophytic fungus is better than organic solvents with higher extraction ratio, safety and lower cost. In vitro testing indicated that the flavonoid extracted by macroporous resin have good antioxidant activity, providing more evidence for the production of flavonoid by biological fermentation method.

## Introduction

Flavonoids are important secondary metabolites of plants, which contain diverse pharmacological activities owning to complex structure types (Yonekura-Sakakibara, Higashi & Nakabayashi, 2019). For example, flavonoids have a strong antioxidant effect on blood circulation and cardiovascular system (Echeverría et al., 2017). Calycosin have significant antiviral activity both in vivo and vitro (Zhu et al., 2009). Most flavonoids have a significant inhibitory effect on the growth of bacteria including *Bacillus subtilis*, *Staphylococcus aureus* and *Escherichia coli* (Kamrani et al., 2007; Gadkowski et al., 2019).

Endophytic fungi widely exist in advanced plant. It has obvious host specificity and tissue specificity (Martin, Romina & Priscila, 2013; Carroll & Carroll, 1978). Endophytic fungi is able to produce the same or similar secondary metabolites of the host, including flavonoid with excellent activity (Qiu et al., 2010; Shih et al., 2017; Shou-Jie et al., 2018; Chi et al., 2019). CBL9 is a flavonoid-producing endophytic fungus from *Conyza blinii* H. Lév, which belongs to *Chaetomium* and is used to produce flavonoid with excellent antioxidant effects in vitro (Tang et al., 2020).

Macroporous resins have been used in the extraction of flavonoids widely as a result of its advantages (Du et al., 2012; Li, Chen & Di, 2012; Chen et al., 2013; Zhang et al., 2018). But macroporous resin has not been used in the endophytic fungi of *Conyza blinii* H. Lév. currently. The macroporous resin D101 was used to extract the total flavonoids of CBL9 for further uses, and the response surface method was optimized to obtain the best process to extract the total flavonoid of the endophytic fungi of *Conyza blinii* H. Lév.

## Materials and Methods

### Plant material

The endophytic fungus CBL9 separated from *Conyza blinii* H. Lév: Biochemistry and Molecular Biology Laboratory of Sichuan Agricultural University preserved

### Chemical reagents

2, 2-Diphenyl-1-picrylhydrazyl (DPPH, HPLC) was purchased from Yuanye Biotechnology Co. (Shanghai, P. R. China). Ferrous Sulfate Heptahydrate (FeSO_4_•7H_2_O), hydrogen peroxide (30%H_2_O_2_), Pyrogallol and Concentrated hydrochloric acid were purchased from Xilong Chemical Co. (Sichuan, P. R. China). Ascorbic acid and Vitamin C (Vc, AR) were purchased from Sinopharm Chemical Reagent Co. (Shanghai, P. R. China). Other chemicals and solvents used in this study were analytical grade.

### Study on optimization of extraction process of crude flavonoid

#### Preparation of raw mater

The flavonoid-producing endophytic fungus CBL9 was inoculated into fresh PDA medium and cultured with shaking at 28 °C until the mycelial pellets grew to a certain condition (Bacteria is not growing and the concentration of flavonoids in the fermentation broth reaches 10 mg/L), then fermentation broth was rotary evaporation at 50 °C and raw material (L9F) was obtained after freeze-drying.

#### Ethyl acetate extraction

The extraction was conducted by following the method of Saraswaty with slight modifications (Saraswaty et al., 2013). A total of 200 mL concentrated fermentation broth was mixed with two times the volume of ethyl acetate to extract the fermentation broth, and repeat extraction three times. Crude flavonoid (L9F-E) was obtained by concentrating by evaporation under freeze-drying.

#### Preparation of standard curve

Sodium nitrite-aluminum nitrate colorimetric method was used to draw rutin standard song by following the method of Tang et al. (2020). The standard curve equation: *A* = 0.4164C-0.0003 (*R*^2^ = 0.999 4).

#### Single factor test

The single factor test method was conducted with slight modifications (Bi & Tan, 2012) and the Macroporous resin D101 was used (Zhang et al., 2018). First, 1.0 g wet resin pretreated was put into 15 conical flasks and mixed with certain L9F. Then the mixture was incubated at different temperature, pH for different time. The adsorption ratio of flavonoid was measured.

#### Response surface analysis

According to the single-factor study, the Box-Benhnken optimization study was designed (Yu et al., 2019) with three levels (adsorption time, temperature, and pH). The adsorption ratio was chosen as inspection index. Design-Expert was used to analyze based on Box-Benhnken data. The possible mathematical model is:

\begin{eqnarray*}Y={\beta }_{0}+\Sigma {\beta }_{\mathrm{i}}{x}_{\mathrm{i}}+\Sigma {\beta }_{\mathrm{ij}}{x}_{\mathrm{i}}{x}_{\mathrm{j}}+\Sigma {\beta }_{\mathrm{ij}}{x}_{\mathrm{i}}^{2} \end{eqnarray*}

Y is the predicted response value; *β*⋅0, *β*_i_, *β*_ii_ and *β*_ij_ is the regression coefficient representing the interaction of intercept, linear, squared and two factors; x_i_ and x_j_ is the independent factor of encoding (i ≠ j).

### Influence of eluent concentration

Gradient volume fraction of ethanol-water solution was used to elute saturated adsorbent at a certain flow rate (1 mL/min). its purity and elution ratio was measured. The total flavonoid (L9F-M) was extracted under optimal condition we got in 2.3.5 and 2.4.

### In vitro antioxidant activity assay

#### DPPH radical scavenging assay

The DPPH radical scavenging activities was collected as previously described in Kaur et al. (2020) and Tsimogiannis & Oreopoulou (2006). Vc was used as the standard antioxidant. L9F was separately dissolved in distilled water to prepare flavonoid solutions of different concentrations (0.01, 0.02, 0.03... 0.09, and 0.10 mg/mL). Equal volume of DPPH solution was mixed with different concentrations of flavonoid solution. The mixture was shaken and then put in a dark place for 30 min. Finally, the absorbance was measured at 517 nm by a spectrophotometer and each experimental group had three parallel controls.

\begin{eqnarray*}Y(\text{%})=[1-({A}_{1}-{A}_{2})/{A}_{3}]\times 100\text{%} \end{eqnarray*}

Y(%) is the DPPH radical scavenging activity A_1_ is the absorbance of the sample with DPPH, A_2_ is the absorbance of the sample without DPPH, and A_3_ is the absorbance of DPPH without the sample.

#### ABTS radical scavenging assay

The ABTS radical scavenging activities was collected as previously described in Kaur and Zhang (Kaur et al., 2020; Zhang, Yang & Zhou, 2018; Zhang et al., 2018). Vc was used as the standard antioxidant. L9F was separately dissolved in distilled water to prepare flavonoid solutions of different concentrations (0.01, 0.02, 0.03 ... 0.09, and 0.10 mg/mL). Two milliliters of ABTS solution was mixed with one hundred Microliters of different flavonoid solution. The mixture incubated in a dark place at room temperature for 6 min. Finally, the absorbance was measured at 734 nm by a spectrophotometer and each experimental group had three parallel controls.

\begin{eqnarray*}Y(\text{%})=[1-({A}_{1}-{A}_{2})/{A}_{3}]\times 100\text{%}, \end{eqnarray*}

Y(%) is the ABTS radical scavenging activity, A_1_ is the absorbance of the sample with ABTS, A_2_ is the absorbance of the sample without ABTS, and A_3_ is the absorbance of ABTS without the sample.

#### Hydroxyl radical (•OH) scavenging assay

The hydroxyl radical scavenging activities of sample was collected as previously described in Chobot (Chobot & Hadacek, 2011), and Vc was used as the standard antioxidant. L9F was separately dissolved in distilled water to prepare flavonoid solutions of different concentrations (0.1, 0.2, 0.3 ... 0.9, and 1.0 mg/mL). A 0.5 mL flavonoid solutions of each concentration was mixed with 1 mL of Salicylic acid (6 mM), 1.5 mL of phosphate buffer solution (PBS, 0.15 M, pH 7.4), 1 mL of ferrous sulfate (6 mM) and 0.5 mL of H_2_O_2_ solution (0.01%). Then, the mixtures were incubated at 37 °C for 30 min. Finally, the absorbance was measured at 510 nm by a spectrophotometer and each experimental group had three parallel controls.

\begin{eqnarray*}Y(\text{%})=[({A}_{1}-{A}_{2})/({A}_{3}-{A}_{2})]\times 100\text{%} \end{eqnarray*}

Y(%) is the •OH radical scavenging activity (%), A_1_ is the absorbance of the sample after reaction with hydroxyl radicals, A_2_ is the absorbance of the sample, and A_3_ is the absorbance without H_2_O_2_.

#### Superoxide radical (•O^2−^) scavenging assay

The superoxide radical scavenging activities of sample was collected as previously described in Zhishen and Leong (Zhishen, Mengcheng & Jianming, 1999; Leong et al., 2008). Similarly, Vc was used as the standard antioxidant. L9F were separately dissolved in distilled water to prepare polysaccharide solutions of different concentrations 0.01, 0.02, 0.03 ... 0.09, and 0.10 mg/mL. A 1.0 mL sample of each concentration was mixed with 3.0 mL of Tris-Hcl buffer (pH 8.2), 0.8 mL of Pyrogallol (0.05 M). Then, the mixtures were bathed at 25 °C for 5 min and 1.0 mL HCl (8.00 M) was mixed after that. Finally, the absorbance was measured at 325 nm using a spectrophotometer.

\begin{eqnarray*}Y(\text{%})=[1-({A}_{1}/{A}_{2})]\times 100\text{%} \end{eqnarray*}

Y is the •O^2−^ radical scavenging activity (%), A_1_ is the absorbance of the sample and A_2_ isthe absorbance of the control.

## Results

### The result of Single factor test

#### The influence of adsorption time

**Figure 1:**
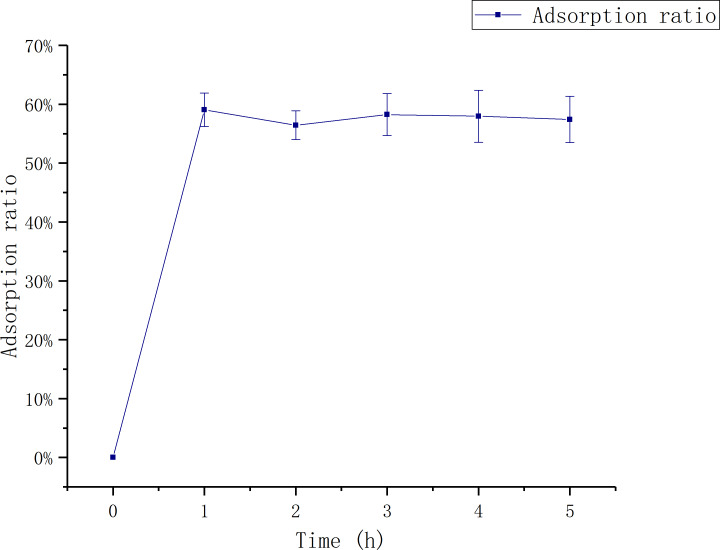
Relationship between adsorption time and adsorption rate. The data point indicates the change of adsorption rate over time.

Figure 1 shows that the adsorption ratio increases first and then stabilizes with the increase of adsorption time. The adsorption ratio reached to the maximum first at 1 h,which was 59.06 ± 0.03%.

#### The influence of pH

**Figure 2:**
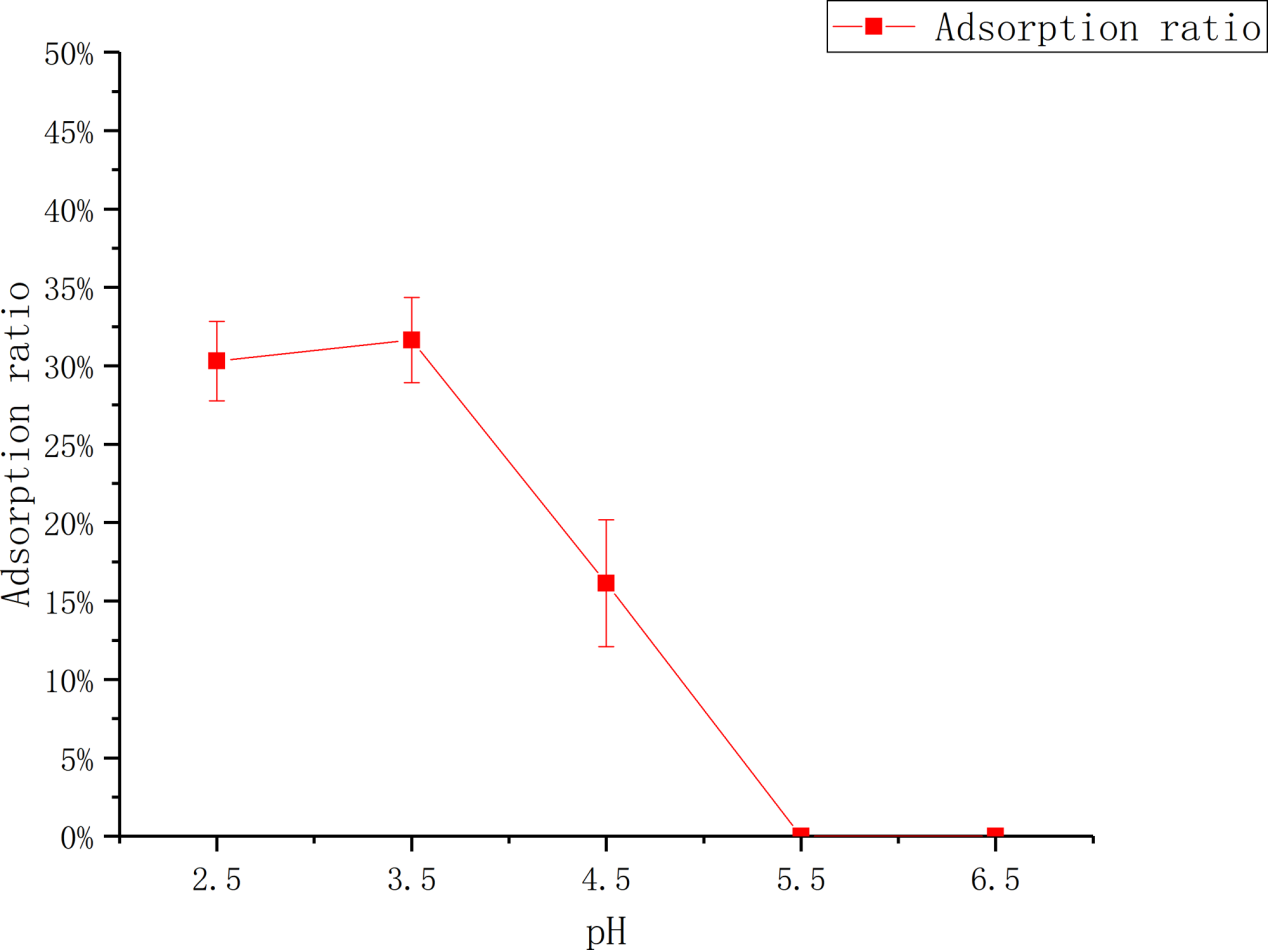
Adsorption of D101 under different pH conditions. The data point indicates the change of adsorption rate over pH.

Figure 2 shows that the adsorption ratio increases first, then goes down with the increase of pH. The adsorption ratio reach to the maximum at pH 3.5, which was (31.65 ± 0.03)%. There was no adsorption when pH was more than 5, which may be caused by the change of the flavonoid structure and the deactivation (Wu et al., 2017) or the weakening of the adsorption capacity of the macroporous resin (Li et al., 2007) under this pH condition.

#### The effect of temperature

**Figure 3:**
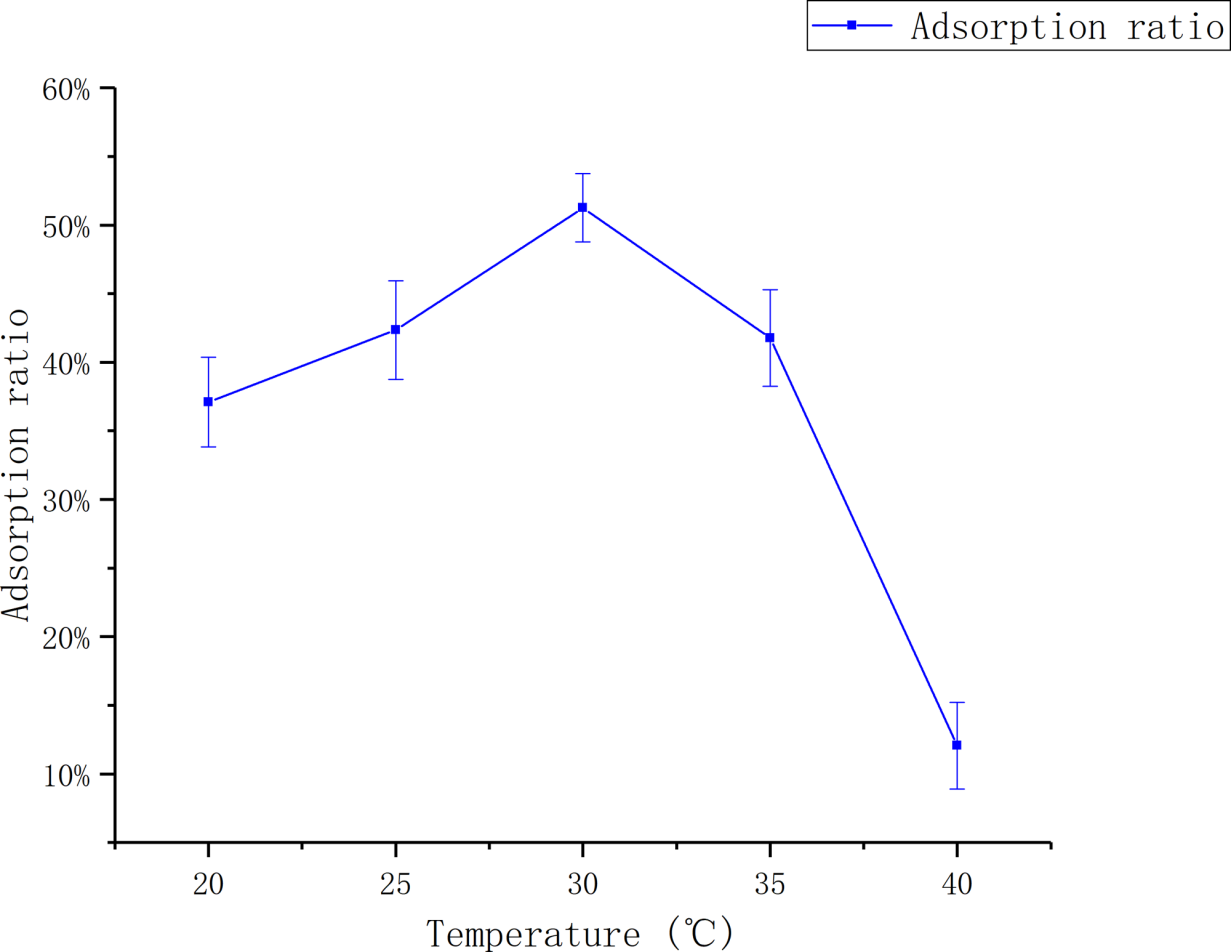
Adsorption of D101 under different liquid temperature. The data point indicates the change of adsorption rate over temperature.

Figure 3 shows that the adsorption ratio increases first and then goes down with the increase of temperature. The adsorption ratio reached to the maximum first at 30 °C, which was 51.26 ± 0.02%.

### Optimization of extraction process by response surface methodology

#### Selection of analysis factor level

According to Box-Benhnken’s central combination test design principle and single-factor test results, three factors (temperature, pH, and time) that have significant effects on the extraction of total flavonoids were selected, and the three-factor three-level response is adopted, which is shown in Table 1.

#### Response surface analysis experiment design scheme

A (temperature), B (pH), and C (adsorption time) was taken as independent variables, and Y (total flavonoid adsorption ratio) was taken as the response value. The test plan and results are shown in Table 2.

#### Establishment and analysis of multivariate quadratic response surface regression model

Quadratic regression response surface analysis Performs in Table 2, and a multiple quadratic response surface regression model was established: *Y* = 0.47-0.056A-0.042B+0.031C+0.088AB-0.043AC+ 4 × 10^−4^BC-0.064A^2^-0.047B^2^+0.056C^2^. The variance analysis of each factor is shown in Table 3.

Table 3 shows that the model is significant (*p* < 0.05) and the Prob>F value of the decisive factor coefficient such as A (temperature), B (pH), C (adsorption time), AB (interaction between temperature and pH), AC (interaction between temperature and adsorption time) are 0.0008, 0.0039, 0.0161, 0.0004, 0.0192 (*p* < 0.05), indicating that the model has a good fit. In addition, the factors affecting the extraction ratio of total flavonoid were A (temperature), B (pH), and C (adsorption time) in order of magnitude, and the temperature reached a significant level (*p* < 0.001).

**Figure 4:**
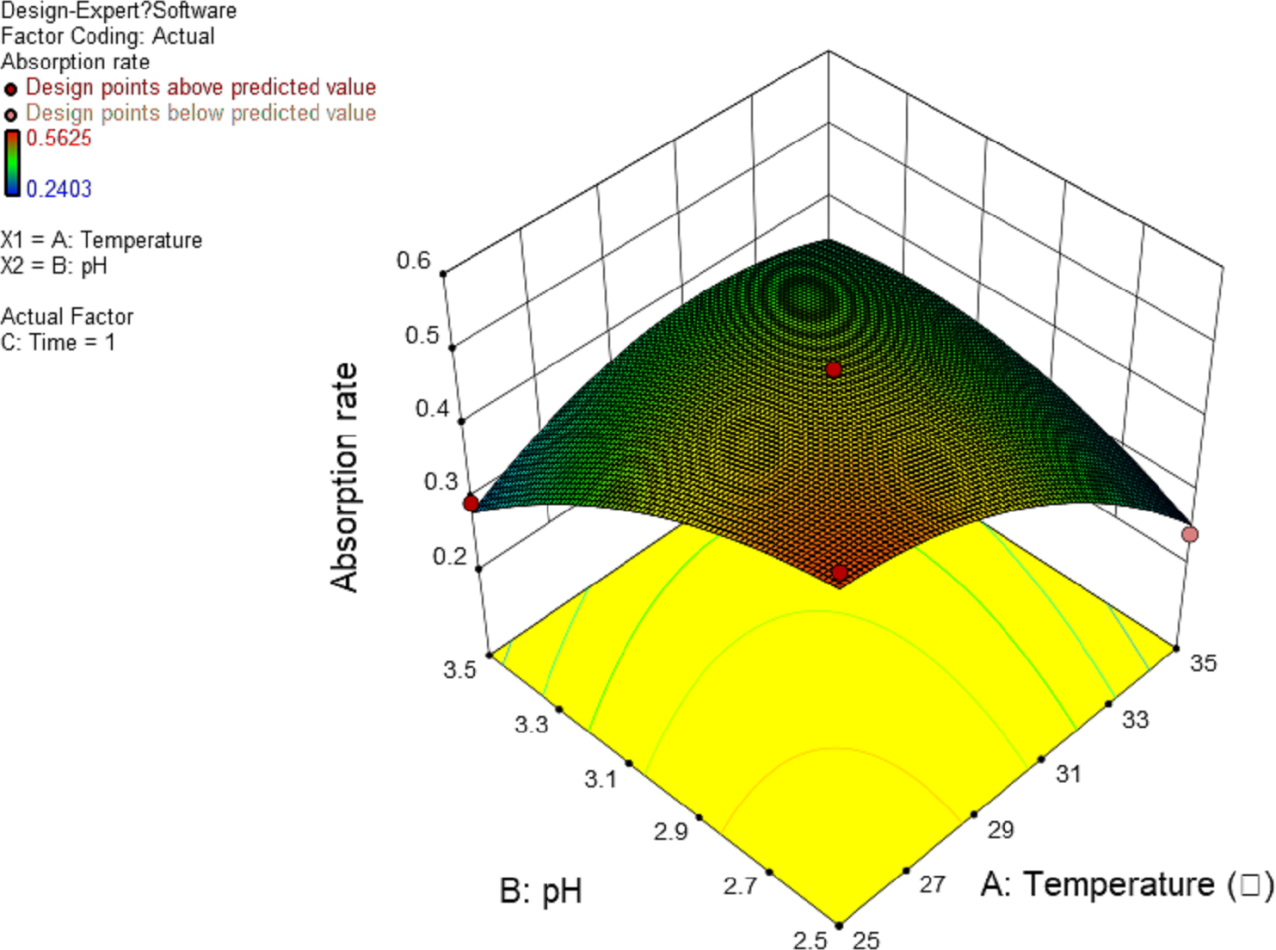
Response surface of interrelated influence of temperature and pH to flavonoids rate.

**Figure 5:**
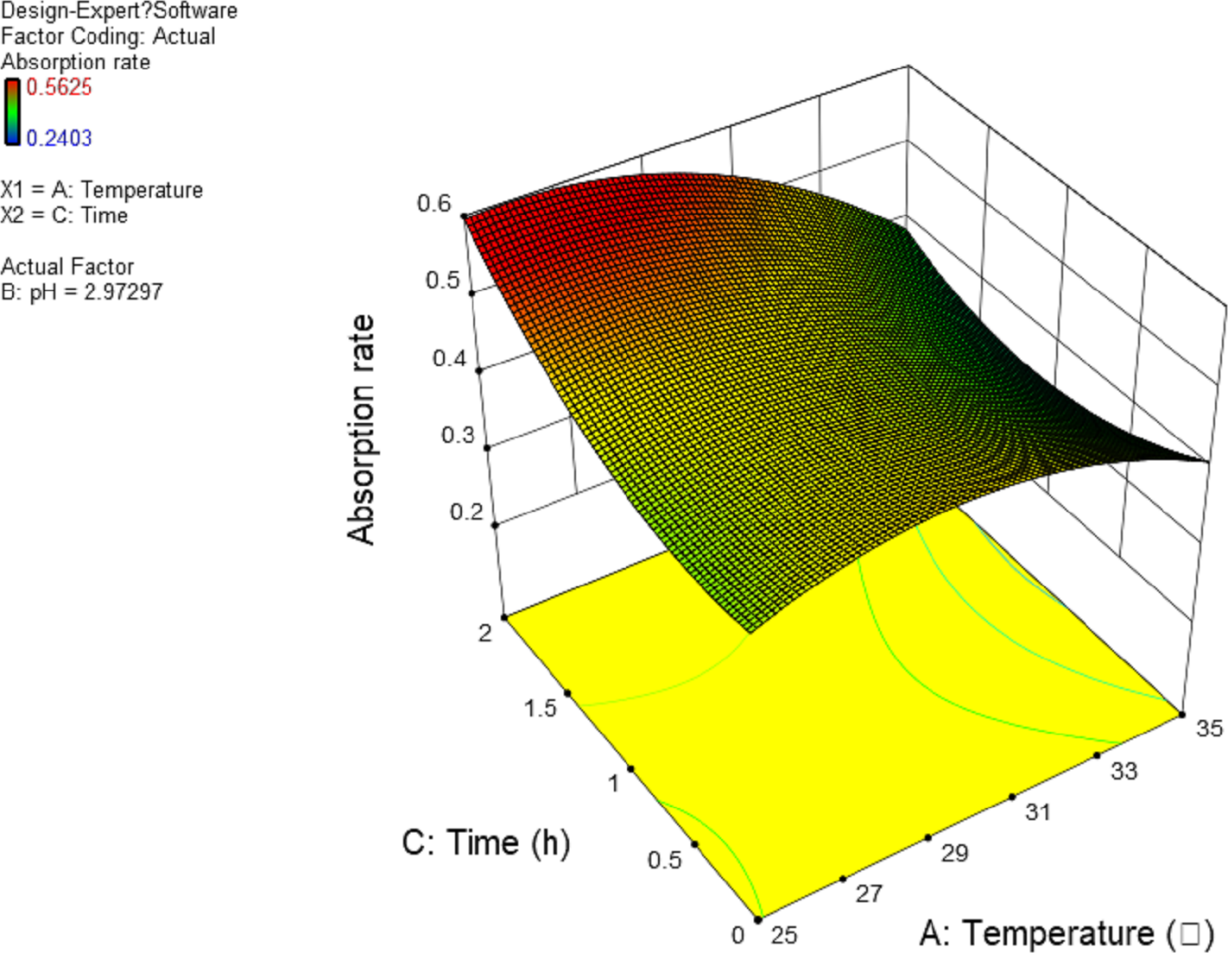
Response surface of interrelated influence of temperature and time to flavonoids rate.

**Figure 6:**
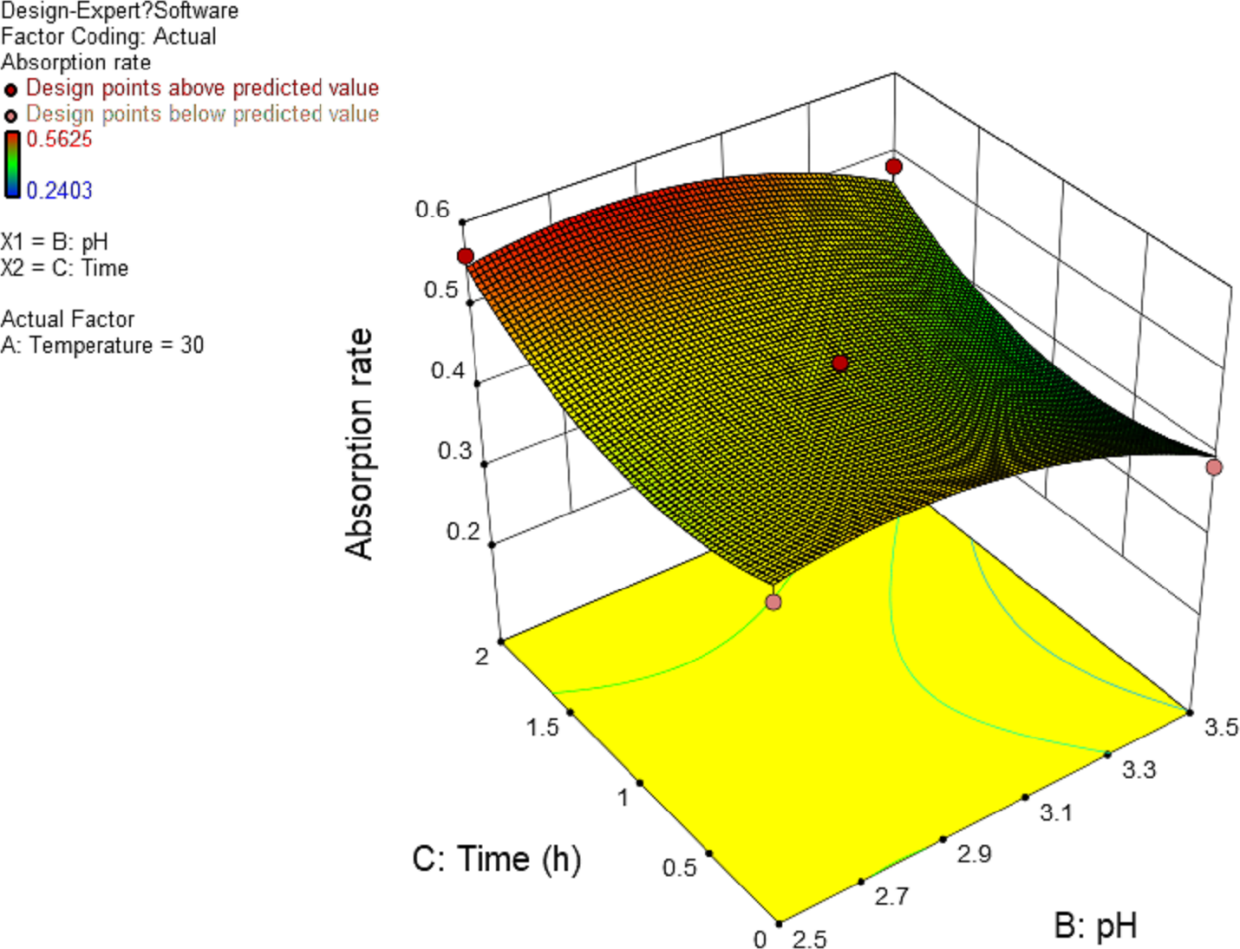
Response surface of interrelated influence of pH and time to flavonoids rate.

In this experiment, the interaction between AB and AC has significant effect. The results are shown in Figs. 4 and 5, and the interaction between BC is shown in Fig. 6. It can be seen from the response surface diagram that the extraction ratio of total flavonoids first increases and then decreases and increases in the end with the increase of A (temperature). B is in the range of −1.0 to 0. Due to the interaction of A, the extraction ratio of total flavonoids is higher. C is in the range of 0 to 1.0, due to the interaction of A, the extraction ratio of total flavonoids is higher.

#### Model verification

It is obtained that the supreme extraction ratio predicted is 64.2% under the process conditions of 25.02 °C, pH 2.80 and 1.85 h by solving the inverse matrix of the quadratic polynomial mathematical model of the total flavonoid yield.

The optimal conditions were revised to 25 °C, pH 2.80, and 1.85 h to extract the total flavonoids by the above to check the validity. The actual adsorption ratio was (64.14 ± 0.04)%, which is close to the theoretical value.

**Table 1:** Factors and the levels of experiment of Response Surface Analysis.

Factors	Factor levels		
	−1	0	1
Temperature/°C	25	30	35
pH	2.5	3.5	4.5
time/h	0	1	2

**Table 2:** Observed and estimated values for different levels of experimental design.

No.	Factors			Adsorption rate/%
	A	B	C	
1	0	0	0	0.4739
2	0	1	1	0.485
3	0	0	0	0.4709
4	1	0	−1	0.448
5	0	−1	−1	0.4667
6	1	1	0	0.3244
7	1	−1	0	0.2403
8	−1	−1	0	0.5625
9	−1	0	−1	0.4396
10	−1	1	0	0.2957
11	0	0	0	0.4739
12	0	0	0	0.4402
13	0	1	−1	0.3885
14	0	−1	1	0.5616
15	−1	0	1	0.555
16	0	0	0	0.4739
17	1	0	1	0.3927

**Table 3:** Analyze of mean square. SS means sum of spuares; DF means degree of freedom; MS means mean square; F means a statistic obtained by analysis of variance based on experimental data; Prob > F means the chance that an F this large could occur due to noise.

Source	SS	DF	MS	F	Prob >F
Model	0.1236	9	0.0137	17.2796	0.0005
A-temperature	0.0250	1	0.0250	31.4807	0.0008
B-pH	0.0142	1	0.0142	17.9143	0.0039
C-time	0.0079	1	0.0079	9.9478	0.0161
AB	0.0308	1	0.0308	38.7302	0.0004
AC	0.0073	1	0.0073	9.1654	0.0192
BC	0.0000	1	0.0000	0.0008	0.9782
A$\hat {}$2	0.0171	1	0.0171	21.5163	0.0024
B$\hat {}$2	0.0093	1	0.0093	11.7547	0.0110
C$\hat {}$2	0.0132	1	0.0132	16.6103	0.0047
Error	0.0056	7	0.0008		
Lack of Fit	0.0047	3	0.0016	7.1415	0.0439
Pure Error	0.0009	4	0.0002		
Total	0.1292	16			

### The effect of eluent concentration

**Figure 7:**
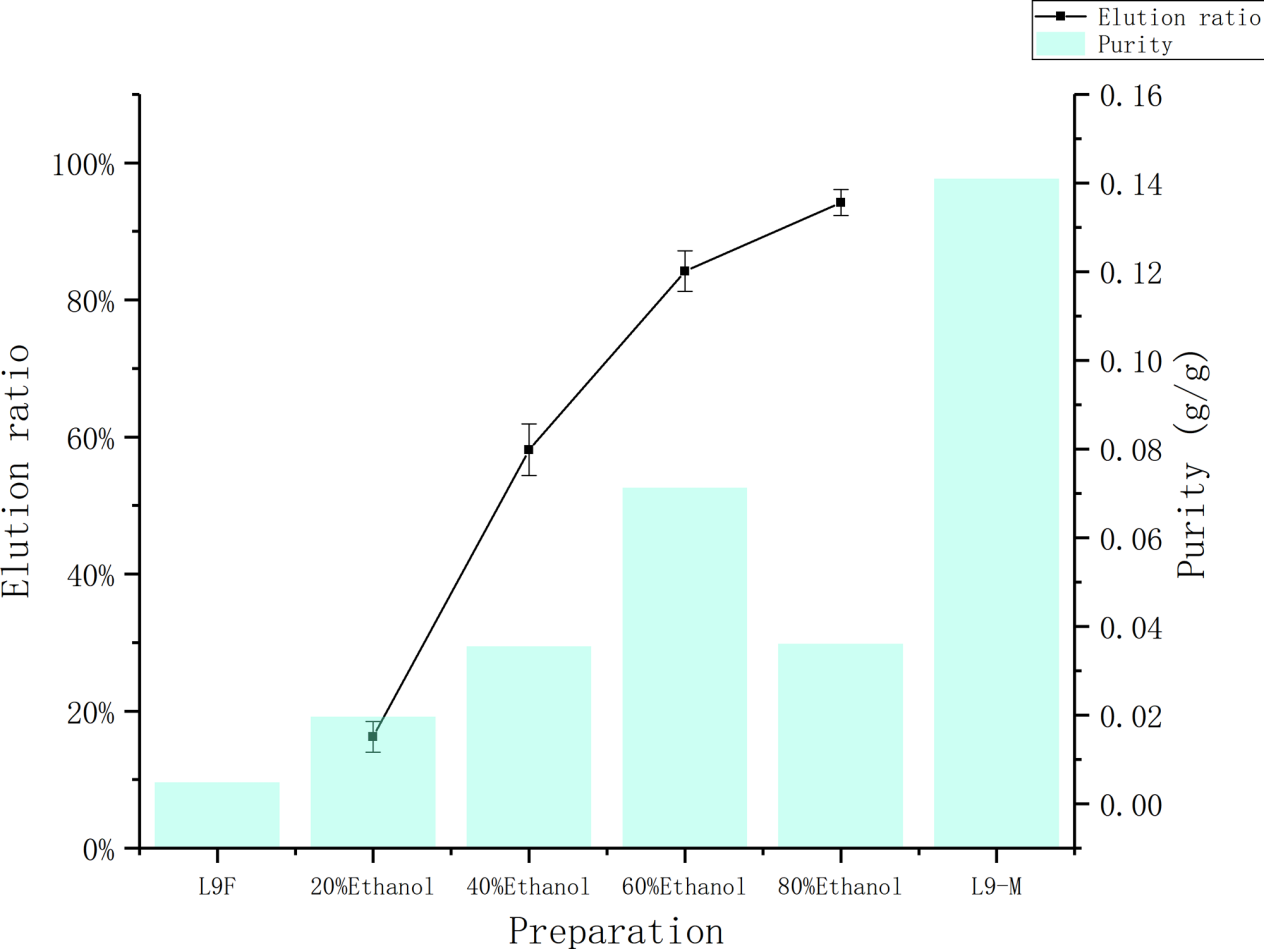
Comparison of elution rate and purity of total flavonoids at different concentrations. The data point indicates the Indicates the change of elution rate and purity of flavonoid over eluent.

Figure 7 shows the ethanol elution effect with different volume fractions. As the volume fraction of the ethanol increases, the elution ratio is increasing. When 60% ethanol was used, the elution ratio of total flavonoids reached to (81.54 ± 0.03)%, and the purity of flavonoids was 7.13%. When 80% ethanol was used, although the elution ratio rises to (90.49 ± 0.03)%, the purity dropped to 3.61%. Therefore 60% ethanol is selected for elution considering the purity and elution ratio.

The total flavonoids prepared by ethyl acetate extraction method, whose purity was 14.1%, but extraction ratio was only (31.68 ± 0.04)%, not only consumes a large amount of raw material and ethyl acetate, but also causes environmental problems. The macroporous resin adsorption method is more economical and environmentally friendly, and the eluate’s purity can rise to 13.69% with precipitated by absolute ethanol.

### In vitro antioxidant activity

#### DPPH radical scavenging assay

**Figure 8:**
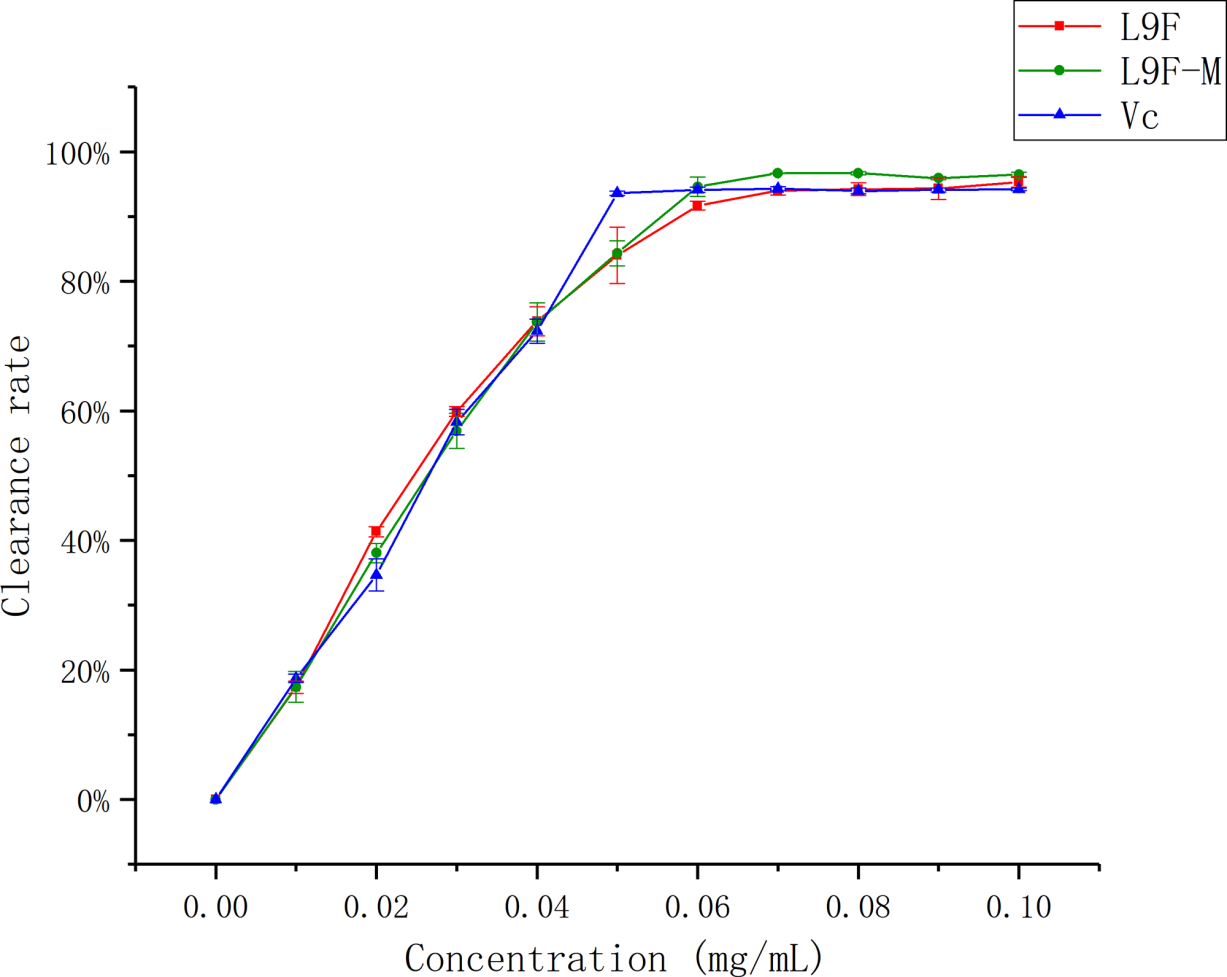
Scavenging ability of total flavonoids of endophytic fungi on DPPH. Each data point indicates the clearance rate of total flavonoids on DPPH before and after extraction, where Vc is the control.

DPPH has been widely accepted as a tool for estimating the free radical scavenging activities of antioxidants (Khled Khoudja, Boulekbache-Makhlouf & Madani, 2014). Figure 8 shows that the DPPH clearance ratio goes up as the concentration of each sample increases and it tends to be flat when the sample mass concentration is greater than 0.05 mg/mL. When the concentration reaches the maximum (0.1 mg/mL), the clearance ratio of total flavonoids on DPPH increases from (95.33 ± 0.01)% to (96.44 ± 0.04)% after purification, and Vc’s clearance ratio is (94.18 ± 0.002)%. It can be seen that the clearance effect of flavonoids on DPPH slightly better than that before purification, and the clearance effect of flavonoids on DPPH is close to Vc.

#### ABTS radical scavenging assay

**Figure 9:**
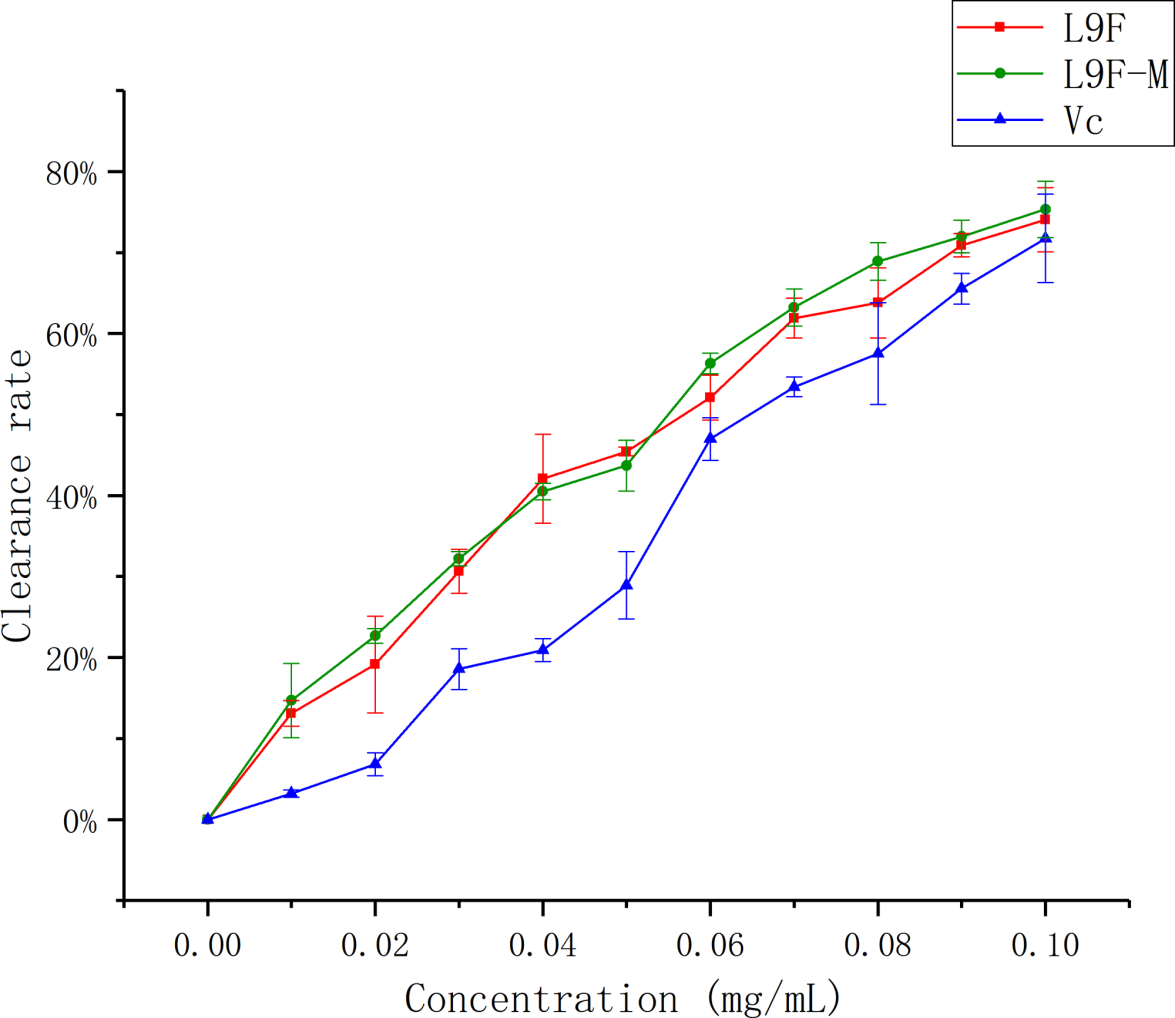
Scavenging ability of total flavonoids of endophytic fungi on ABTS. Each data point indicates the clearance rate of total flavonoids on ABTS before and after extraction, where Vc is the control.

ABTS has been widely accepted as a tool for estimating the free radical scavenging activities of antioxidants (Thaipong et al., 2012). Figure 9 shows that the ABTS clearance ratio goes up as the mass concentration of each sample increases. When the concentration reaches the maximum (0.1 mg/mL), the clearance ratio of total flavonoids on ABTS increases from (74.06 ± 0.04)% to (75.33 ± 0.03)% after purification, and Vc’s clearance ratio is (71.74 ± 0.05)%. It can be seen that the clearance effect of flavonoid on ABTS slightly better than that before purification, and the clearance effect of flavonoid on ABTS is better than Vc.

#### Hydroxyl radical (•OH) scavenging assay

**Figure 10:**
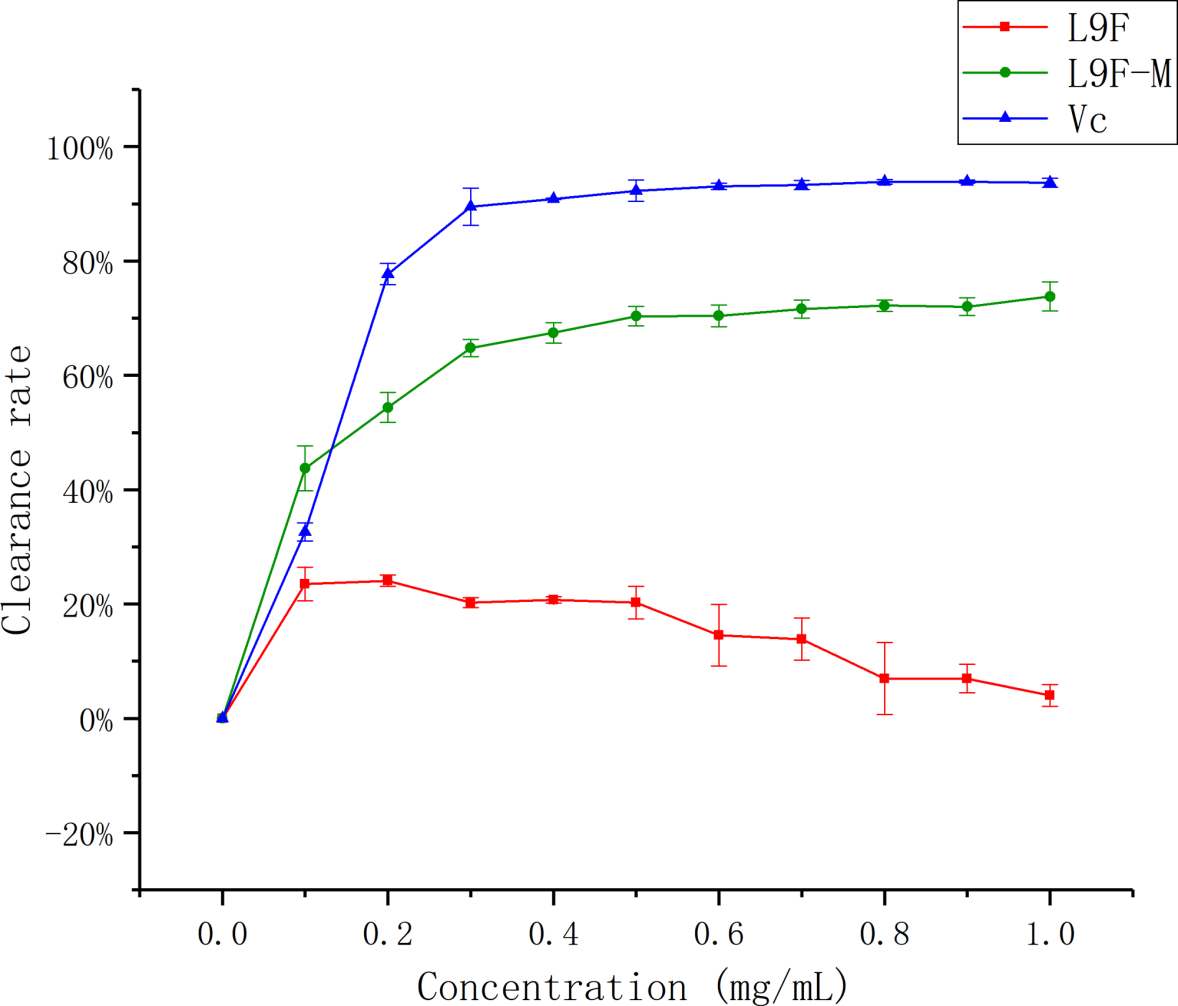
Scavenging ability of total flavonoids of endophytic fungi on −OH. Each data point indicates the clearance rate of total flavonoids on −OH before and after extraction, where Vc is the control.

Hydroxyl radicals are very active and have been associated with cancer risk when accumulated in the body excessively (Sakanaka, Tachibana & Okada, 2005). Figure 10 shows that the •OH clearance ratio goes up as the mass concentration of each sample increases and it tends to be flat when the sample mass concentration is greater than 0.3 mg/mL, but the clearance ratio of the sample before purification shows a downward trend, which may be the presence of oxidized and discolored impurities in the original fermentation broth. When the sample concentration reaches the maximum (1 mg/mL), the clearance ratio of total flavonoids on •OH increases from (3.98 ± 0.02)% to (73.79 ± 0.02)% after purification, and Vc’s clearance ratio is (93.72 ± 0.01)%. It can be seen that the clearance effect of flavonoids on •OH better than that before purification.

#### Superoxide radical (•O^2−^) scavenging assay

**Figure 11:**
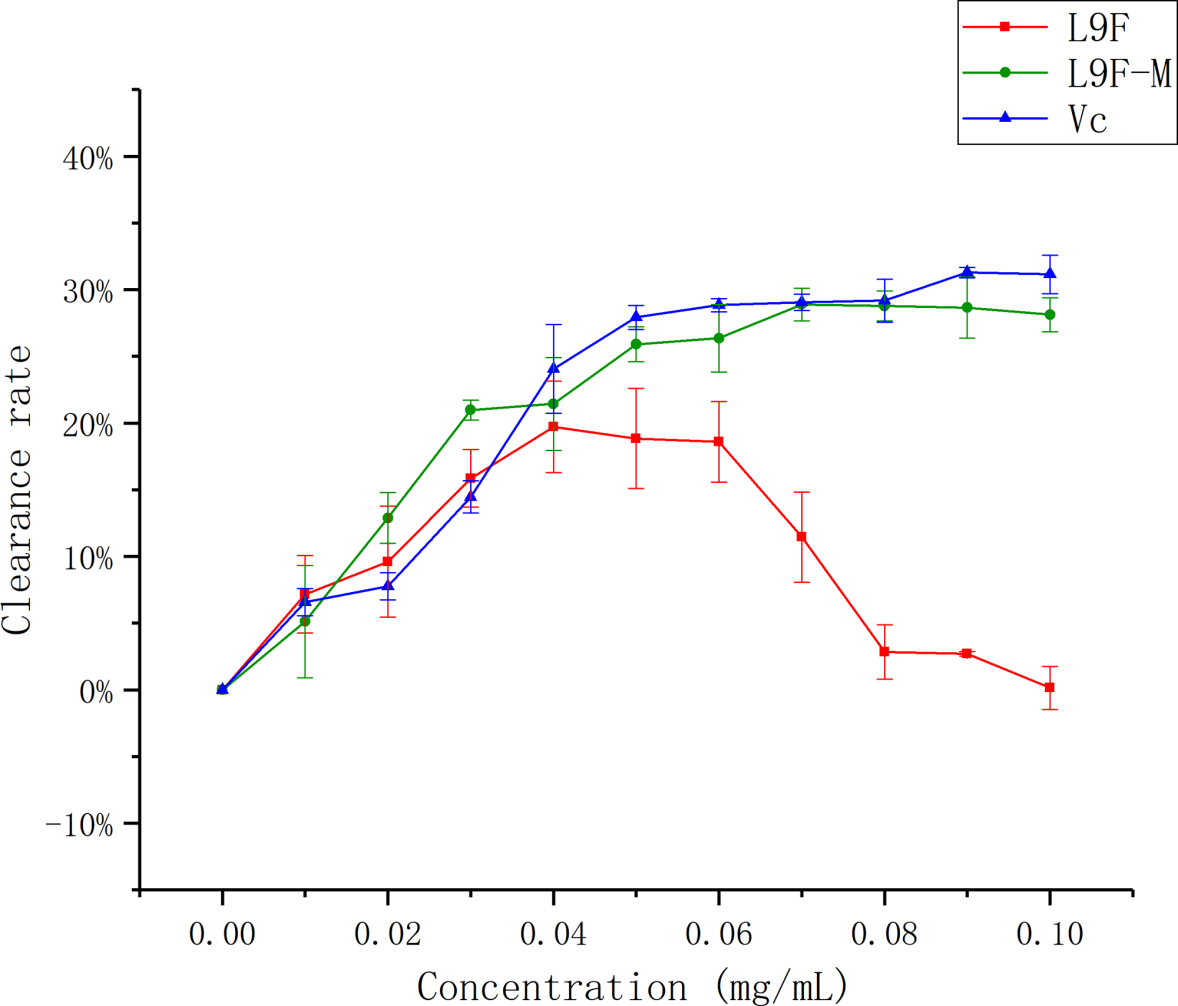
Scavenging ability of total flavonoids of endophytic fungi on •*O*^2−^. Each data point indicates the clearance rate of total flavonoids on •*O*^2−^ before and after extraction, where Vc is the control.

The superoxide radical is known to be very harmful to cellular components as a precursor of more reactive oxygen species, contributing to tissue damage and various diseases (Ozsoy et al., 2008; Aruoma, Grootveld & Bahorun, 2006). Figure 11 shows that the •O^2−^ clearance ratio goes up as the mass concentration of each sample increases and it tends to be flat when the sample mass concentration is greater than 0.05 mg/mL, but the clearance ratio of the sample before purification shows a downward trend, which may be the presence of oxidized and discolored impurities in the original fermentation broth. When the sample concentration reaches the maximum (0.1 mg/mL), the clearance ratio of total flavonoids on •O^2−^ increases from (0.15 ± 0.016)% to (28.11 ± 0.01)% after purification, and Vc’s clearance ratio is (31.14 ± 0.01)%. It can be seen that the clearance effect of flavonoids on •O^2−^ better than that before purification.

## Discussion

Flavonoids have attracted more and more attention because of their complex structure and function over these years (Baran et al., 2020; Zou et al., 2020; Zhang et al., 2020). However, flavonoids from plants are mainly used in the market now. The regeneration ratio is affected by the natural growth of plants, and the yield is limited. Therefore, the objective of this study was to extract flavonoid by macroporous resin and analyse antioxidant activities.

It has been shown that the extraction procedure has a significant impact on the yield and structural characteristics of flavonoids, as well as their biological activities (Taghinia, Khodaparast & Ahmadi, 2019). The macroporous resin adsorption method has been widely used in the extraction and purification of total flavonoid from plants (Du et al., 2012; Zhang et al., 2018). The macroporous resin adsorption method is more economical and environmentally friendly with similar purity and higher extraction ratio compared with commonly used organic solvent method.

It has been found that the generation of reactive oxygen species (ROS) and the corresponding response to oxidative stress are critical factors in the outbreak of several human diseases (Lee & Lee, 2006). Antioxidants have vital functions against ROS in the biological system (Zhang et al., 2015). In the present study, the antioxidant activity of flavonoid was studied by DPPH, ABTS, superoxide radical and hydroxyl radicals. The results showed that flavonoids exhibited stronger antioxidant activity than against DPPH, ABTS, superoxide radical and hydroxyl radicals, and the clearance ratio is highly closed to total flavonoid isolated by ethyl acetate (Tang et al., 2020).

These results indicated that the total flavonoid extracted from the endophytic fungus L9 from *Conyza blinii* H. Lév by macroporous resin has good antioxidant activity, which is closed to ethyl acetate extraction. The method with macroporous resin is better than which with ethyl acetate in many aspects. Further scientific work in our laboratory is in progress to separate it.

## Conclusions

In the present study, we used the response surface method to optimize the extraction of total flavonoids from the endophytic fungus L9 from *Conyza blinii* H. Lév by macroporous resin for the first time. It was found that the best adsorption ratio reached (64.14 ± 0.04)% and the purity of the total flavonoids was increased by 14.55 times compared with the original fermentation broth. In the antioxidant research, L9F-M has good antioxidant activity in vitro. These results demonstrate that macroporous resin in the extraction of total flavonoids from endophytic fungus is better than organic solvents with higher extraction ratio, safety, lower cost and good antioxidant, which provides more evidence for the production of flavonoids by biological fermentation method. However, the further applications remain to be explored in future studies.

##  Supplemental Information

10.7717/peerj.11223/supp-1 File S1 The dataset of the single factors and anti-oxidation The single factor is showing the absorbance and absorbance rate of the sample in related experiments. These data are used to compare the changes in the purity and activity of total flavonoids before and after extraction. The anti-oxidation is showing the clearance of the sample to DPPH ABTS Hydroxyl and Superoxide.

## Additional Information and Declarations

### Data Availability

The following information was supplied regarding data availability: The raw measurements are available in the Supplemental File.

## References

[ref-1] Aruoma OI, Grootveld M, Bahorun T (2006). Free radicals in biology and medicine: from inflammation to biotechnology. Biofactors.

[ref-2] Baran MY, Emecen G, Simon A, Tóth G, Kuruuzum-Uz A (2020). Assessment of the antioxidant activity and genotoxicity of the extracts and isolated glycosides with a new flavonoid from Lotus aegaeus (Gris.) Boiss. Industrial Crops and Products.

[ref-3] Bi YG, Tan YQ (2012). Study on macroporous resin separation and purification of total flavonoids of plantago process. Advanced Materials Research.

[ref-4] Carroll GC, Carroll FE (1978). Studies on the incidence of coniferous needle endophytes in the Pacific Northwest. Canadian Journal of Botany.

[ref-5] Chen Z, Long J, Kang L, Du X, Di D (2013). Modified macroporous adsorption resin (LX1180) used to adsorb flavonoid. Pigment & Resin Technology.

[ref-6] Chi WC, Pang KL, Chen WL, Wang GJ, Lee TH (2019). Antimicrobial and iNOS inhibitory activities of the endophytic fungi isolated from the mangrove plant *Acanthus ilicifolius* var. xiamenensis. Botanical Studies.

[ref-7] Chobot V, Hadacek F (2011). Exploration of pro-oxidant and antioxidant activities of the flavonoid myricetin. Redox Report.

[ref-8] Du H, Wang H, Yu J, Liang C, Li P (2012). Enrichment and purification of total flavonoid C-glycosides from abrus mollis extracts with macroporous resins. Industrial & Engineering Chemistry Research.

[ref-9] Echeverría J, Julia O, Leonora M, Urzúa A, Marcela W (2017). Structure-activity and lipophilicity relationships of selected antibacterial natural flavones and flavanones of *Chilean Flora*. Molecules.

[ref-10] Gadkowski W, Siepka M, Janeczko T, Kostrzewa-Susow E, Popoński J, Mazur M, Arowska B, Aba W, Maciejewska G, Wawrzeńczyk C (2019). Synthesis and antimicrobial activity of methoxy-substituted *γ*-Oxa-ε-lactones derived from flavanones. Molecules.

[ref-11] Kamrani YY, Amanlou M, Esmaeelian B, Bidhendi SM, Sahebjamei M (2007). Inhibitory effects of a flavonoid-rich extract of pistacia vera hull on growth and acid production of bacteria involved in dental plaque. International Journal of Pharmacology.

[ref-12] Kaur N, Arora DS, Kalia N, Laur M (2020). Antibiofilm, antiproliferative, antioxidant and antimutagenic activities of an endophytic fungus *Aspergillus fumigatus* from Moringa oleifera. Molecular Biology Reports.

[ref-13] Khled Khoudja N, Boulekbache-Makhlouf L, Madani K (2014). Antioxidant capacity of crude extracts and their solvent fractions of selected Algerian Lamiaceae. Industrial Crops and Products.

[ref-14] Lee KW, Lee HJ (2006). Biphasic effects of dietary antioxidants on oxidative stress-mediated carcinogenesis. Mechanisms of Ageing & Development.

[ref-15] Leong CNA, Tako M, Hanashiro I, Tamaki H (2008). Antioxidant flavonoid glycosides from the leaves of *Ficus pumila* L. Food Chemistry.

[ref-16] Li J, Chen Z, Di D (2012). Preparative separation and purification of Rebaudioside A from Stevia rebaudiana Bertoni crude extracts by mixed bed of macroporous adsorption resins. Food Chemistry.

[ref-17] Li YM, Gao J, Yang ZJ, Lin ML (2007). Studt on the adsorbing and refining alkaloid from *Cynanchumkomaroviial.Iljinski* with macro-porous resin. Journal of Inner Mongolia University of Technology.

[ref-18] Martin U, Romina G, Priscila C (2013). Endophytic fungi from Peruvian highland and lowland habitats form;distinctive and host plant-specific assemblages. Biodiversity & Conservation.

[ref-19] Ozsoy N, Can A, Yanardag R, Akev N (2008). Antioxidant activity of Smilax excelsa L. leaf extracts. Food Chemistry.

[ref-20] Qiu M, Xie RS, Shi Y, Zhang H, Chen HM (2010). Isolation and identification of two flavonoid-producing endophytic fungi from *Ginkgo biloba* L. Annals of Microbiology.

[ref-21] Sakanaka S, Tachibana Y, Okada Y (2005). Preparation and antioxidant properties of extracts of Japanese persimmon leaf tea (kakinoha-cha). Food Chemistry.

[ref-22] Saraswaty V, Srikandace Y, Simbiyani NA, Jasmansyah P, Udin Z (2013). Antioxidant activity and total phenolic content of endophytic fungus *Fennellia nivea* NRRL 5504. Pakistan Journal of Biological ences Pjbs.

[ref-23] Shih TC, Tian X, Huang C, Huang J (2017). Identification of flavonoids and flavonoid-producing endophytic fungi isolated from *opisthopappus*. Bangladesh Journal of Botany.

[ref-24] Shou-Jie L, Xuan Z, Xiang-Hua W, Chang-Qi Z (2018). Novel natural compounds from endophytic fungi with anticancer activity. European Journal of Medicinal Chemistry.

[ref-25] Taghinia P, Khodaparast MHH, Ahmadi M (2019). Free and bound phenolic and flavonoid compounds of Ferula persica obtained by different extraction methods and their antioxidant effects on stabilization of soybean oil. Journal of Food Measurement and Characterization.

[ref-26] Tang Z, Wang Y, Yang J, Xiao Y, Wang G (2020). Isolation and identification of flavonoid-producing endophytic fungi from medicinal plant *Conyza blinii* H.Lév that exhibit higher antioxidant and antibacterial activities. PeerJ.

[ref-27] Thaipong K, Boonprakob U, Crosby K, Cisneros-Zevallos L, Byrne DH (2012). Comparison of ABTS, DPPH, FRAP, and ORAC assays for estimating antioxidant activity from guava fruit extracts. Journal of Food Composition & Analysis.

[ref-28] Tsimogiannis DI, Oreopoulou V (2006). The contribution of flavonoid C-ring on the DPPH free radical scavenging efficiency. A kinetic approach for the 3′, 4′-hydroxy substituted members. Innovative Food science & Emerging Technologies.

[ref-29] Wu DQ, Ma ZY, Hei JW, Li S (2017). Antioxidant stability of flavonoid from oriental stephania root. West China Journal of Pharmaceutical Sciences.

[ref-30] Yonekura-Sakakibara K, Higashi Y, Nakabayashi R (2019). The origin and evolution of plant flavonoid metabolism. Frontiers in Plant Science.

[ref-31] Yu M, Wang B, Qi Z, Xin G, Li W (2019). Response surface method was used to optimize the ultrasonic assisted extraction of flavonoids from *Crinum asiaticum*. Saudi Journal of Biological Sciences.

[ref-32] Zhang CH, Yu Y, Liang YZ, Chen XQ (2015). Purification, partial characterization and antioxidant activity of polysaccharides from Glycyrrhiza uralensis. International Journal of Biological Macromolecules.

[ref-33] Zhang H, Yang YF, Zhou ZQ (2018). Phenolic and flavonoid contents of mandarin (Citrus reticulata Blanco) fruit tissues and their antioxidant capacity as evaluated by DPPH and ABTS methods. Journal of Integrative Agriculture.

[ref-34] Zhang L, Wu T, Wang Z, Ding G, Zhao L (2018). Enrichment and purification of total ginkgo flavonoid O-glycosides from *ginkgo biloba* extract with macroporous resin and evaluation of anti-inflammation activities in vitro. Molecules.

[ref-35] Zhang XH, Shen J, Zhao CC, Shao JH (2020). A new flavonoid glycoside with *α*-glucosidase inhibitory activity from *Galium Verum*. Chemistry of Natural Compounds.

[ref-36] Zhishen J, Mengcheng T, Jianming W (1999). The determination of flavanoid contents on mulberry and their scavenging effects on superoxide radical. Food Chemistry.

[ref-37] Zhu H, Zhang Y, Ye G, Li Z, Zhou P, Huang C (2009). In vivo and in vitro antiviral activities of Calycosin-7-O- *β*-D-glucopyranoside against Coxsackie virus B3. Biological & Pharmaceutical Bulletin.

[ref-38] Zou Y, Xin X, Xu H, Yuan H, Zhao G (2020). Highly efficient bioconversion of flavonoid glycosides from citrus-processing wastes in solvent-buffer systems. Green Chemistry.

